# Lingo1 in the hippocampus contributes to cognitive dysfunction after anesthesia and surgery in aged mice

**DOI:** 10.7150/ijbs.98376

**Published:** 2025-01-01

**Authors:** Changliang Liu, Changteng Zhang, Ling Chen, Xin Liu, Jiahui Wu, Yalan Sun, Jin Liu, Chan Chen

**Affiliations:** 1 Department of High Altitude Medicine, and Center for High Altitude Medicine, West China Hospital, Sichuan University, Chengdu, Sichuan 610041, China.; 2 High Altitude Medicine Key Laboratory of Sichuan Province, Institute of High Altitude Medicine, West China Hospital, Sichuan University, Chengdu, Sichuan 610041, China.; 3 Department of Anesthesiology, West China Hospital, Sichuan University, Chengdu, Sichuan 610041, China.; 4 National-Local Joint Engineering Research Centre of Translational Medicine of Anesthesiology, West China Hospital, Sichuan University, Chengdu, Sichuan 610041, China.; 5 Laboratory of Pulmonary Immunology and Inflammation, Frontiers Science Center for Disease-Related Molecular Network, West China Hospital, Sichuan University, Chengdu 610041, China.

**Keywords:** postoperative cognitive dysfunction, myelin loss, abnormal phosphorylation of the tau protein, neuronal apoptosis

## Abstract

Cognitive impairment caused by anesthesia and surgery is one of the most common complications with multiple etiologies that occurs in elderly patients. The underlying mechanisms are not fully understood, and there is a lack of therapeutic strategies. Increasing evidence has demonstrated that myelin loss, abnormal phosphorylation of the tau protein and neuronal apoptosis are substantial driving factors of cognitive deficits. However, the key regulatory factors involved in the pathology of postoperative cognitive dysfunction require further investigation. Herein, we identified a key regulator, Lingo1, whose expression significantly increased in hippocampal neurons after aged mice underwent unilateral nephrectomy. Elevated Lingo1 expression markedly activated the RhoA/ROCK1 signaling pathway through interactions with NgR and p75NTR, subsequently promoting myelin loss and abnormal phosphorylation of the tau protein. Moreover, the upregulation of Lingo1 in hippocampal neurons further inhibited the EGFR/PI3K/Akt pathway, which may increase neuronal apoptosis. These pathological changes ultimately lead to cognitive impairment in aged mice after surgery. Notably, Lingo1 knockdown significantly reversed pathological changes in the hippocampus and attenuated cognitive decline. In conclusion, our findings highlight that Lingo1 upregulation in hippocampal neurons promotes the occurrence and development of postoperative cognitive dysfunction by regulating myelin loss, abnormal tau phosphorylation and neuronal apoptosis, suggesting that Lingo1 might be a potential target for treating postoperative cognitive dysfunction.

## 1. Introduction

Postoperative cognitive dysfunction (POCD) affects an increasing number of surgical patients as a common neurological complication, and the prevalence of POCD is particularly high in elderly patients undergoing major surgery^1^-^3^. The occurrence of POCD is usually associated with longer hospital stays, impaired activities of daily living, poor prognosis and increased mortality; it also severely places a large financial burden on families and health systems^4^-^6^. The occurrence of POCD depends on various factors, such as patient age, anesthesia method, surgical procedure, occurrence of postsurgical pain and individual factors. Specifically, patient age has been suggested to be the major primary susceptibility factor for POCD^6^, ^7^. In addition, pathological processes such as neuroinflammation^8^, apoptosis^9^, oxidative stress^10^, microglial activation^11^, impairment of synaptic plasticity^12^, destruction of the blood-brain barrier^13^, abnormal tau phosphorylation and accumulation of β-amyloid protein^14^ are closely related to the occurrence and development of POCD. Although enormous efforts have been made to clarify the etiology of cognitive deficits caused by surgery under anesthesia in recent decades, the key mechanism involved is still not fully understood.

Cognitive impairment resulting from surgery under anesthesia shows significant similarities with early-stage AD in terms of clinical manifestations, pathological processes and biomarkers, suggesting a potential common pathogenesis between AD and POCD^7^, ^15^-^17^. Notably, the expression of the myelination-related gene *Lingo1* was found to be significantly perturbed in both neuronal and glial cells during the pathological process of AD according to a previous single-cell transcriptomic analysis^18^, indicating that Lingo1 might be a potential therapeutic target for AD. However, whether *Lingo1* is involved in the occurrence and development of POCD and the potential regulatory mechanism remain uncertain.

Lingo1 is a transmembrane leucine-rich protein encoded by the LRRN6A gene, which is located on chromosome 15q24.3^19^. It contains an immunoglobulin and a leucine-rich repeat domain and is selectively expressed in the spinal cord and brain^19^, ^20^. Increasing evidence has confirmed that Lingo1 plays substantial roles in regulating brain development and neurological disorders, acting as an important negative regulator of neuronal survival, oligodendrocyte differentiation, axonal regeneration and myelination^20^. Lingo1 signal transduction generally relies on the formation of receptor complexes with NgR and p75NTR^21^. It has an expression profile similar to that of NgR, with the lowest levels in the spinal cord and the highest levels in the cerebral cortex. Nevertheless, the direct binding partner of Lingo1 and NgR, p75NTR, is widely expressed in the brain^21^. Interestingly, Lingo1, NgR and p75NTR were found to be significantly upregulated in postmortem brain tissue from AD patients^22^-^24^. Antagonists of Lingo1 can ameliorate cognitive deficits by inhibiting its expression or function^23^, ^25^. These findings suggest that Lingo1 has significant potential value in treating neurological diseases, including cognitive dysfunction caused by surgery under anesthesia or AD pathology.

Lingo1 is specifically expressed in both oligodendrocytes and neurons as a key regulator of myelination and plays essential roles in regulating neuronal development and health through the formation of receptor complexes with NgR/p75NTR or NgR/TORY^19^, ^20^. The myelin sheath is an insulating layer with a thickness proportional to the diameter of the axon^26^. The formation and good structural integrity of the myelin sheaths that wrap axons ensure the rapid propagation of electrical impulses constitute the biological basis of signal transduction in neurons and are prerequisites for learning and memory^27^, ^28^. However, normal myelin structure and function are disrupted by aging and neurodegenerative diseases. Previous functional imaging and histological studies of AD patients have demonstrated remarkable alterations in the structure of white matter, which is composed of axons and myelin^27^. Moreover, a considerable decrease in myelin generation, which has been proven to contribute to a decline in cognitive function, was also observed during aging^29^. Although compensatory hypermyelination is observed following demyelination during the pathological progression of cognitive impairment, hypertrophic myelin sheaths are generally uncompacted and unravel protrusions, and myelin hypertrophy leads to a marked loss of myelin integrity^26^, ^29^, ^30^. These myelin changes have been confirmed to result in cognitive dysfunction in both elderly patients and nonhuman primates^31^.

In addition, abnormal phosphorylation of the tau protein has been proven to be an important pathogenic factor in POCD^32^, ^33^. A previous report showed that the exposure of P6 mice to sevoflurane significantly induced the phosphorylation of the tau protein, abnormal activation of GSK3β and cognitive dysfunction. However, both tau phosphorylation and cognitive decline were alleviated by the administration of an inhibitor of GSK3β or by the knockout of the tau protein-encoding gene MAPT, indicating that abnormal tau phosphorylation might be an important inducer of POCD^33^, ^34^. Notably, increasing evidence has revealed that signaling molecules downstream of Lingo1 might be associated with the abnormal phosphorylation of the tau protein^35^-^37^. Nevertheless, it remains to be further determined whether and how Lingo1 is involved in abnormal tau phosphorylation during POCD. Therefore, we established an aged mouse model of POCD through unilateral nephrectomy under isoflurane anesthesia. Decreases in the spontaneous alternation rate in the Y maze and the recognition index in the novel object recognition (NOR) test indicated significant learning and memory impairment after anesthesia and surgery. In addition, substantial upregulation of Lingo1 was observed in hippocampal neurons in the CA1, CA2 and CA3 subregions after anesthesia and surgery, which resulted in cognitive dysfunction through the promotion of myelin loss, abnormal tau phosphorylation and neuronal apoptosis. When the expression of Lingo1 in hippocampal neurons was knocked down by shRNA(Lingo1), myelin sheath loss, tau phosphorylation and apoptosis were dramatically reversed, further attenuating surgery-induced cognitive impairment, demonstrating that Lingo1 might be a potential therapeutic target for the clinical treatment of POCD.

## 2. Materials and Methods

### 2.1 Animals

Fifteen-month-old male mice purchased from Chengdu Dossy Experimental Animals Co., Ltd., were housed in specific-pathogen-free (SPF) grade feeding rooms with a circadian rhythm of twelve hours at an ambient temperature of 22 ± 1℃ and a relative humidity of 60 ± 10%. All the mice were allowed free access to food and water. All the animal experimental protocols strictly adhered to the Guidelines for Care and Use of Laboratory Animals of the National Institute of Health and were authorized by the Animal Care and Use Committee of Sichuan University.

### 2.2 Establishment of the mouse model

The aged mouse model of POCD was established *via* unilateral nephrectomy under isoflurane according to our previous work^8^. In brief, the mice were placed on the operating table in the right lateral position after being anesthetized with 3% isoflurane (RWD Life Science, Shenzhen, China). Afterward, the isoflurane concentration was adjusted to 1.5% to maintain anesthesia, and an approximately one-centimeter incision was made under the costal margin. After 5 min of exploration of the abdominal organ, the left kidney was removed. The peritoneum and skin were sewn with sterile 4-0 nylon sutures. Postoperative analgesia was achieved by injecting 50 μL of 2% lidocaine (J&K Scientific, Beijing, China) subcutaneously. Thereafter, the mice were transferred to the feeding room after recovery on a heating pad. The mice in the control group did not undergo anesthesia or surgery.

### 2.3 Neurobehavioral tests

Cognitive function and locomotor activity of the mice after unilateral nephrectomy were investigated through open field tests (OFTs), Y maze tests and novel object recognition (NOR) tests. The mice were allowed to adapt to the experimental environment for at least 60 min before testing, and the odors in the experimental chamber were eliminated with 75% alcohol.

OFTs: The mice were gently placed individually in an open field chamber (40 × 40 × 40 cm, RWD Life Science, Shenzhen, China) and allowed to explore freely for five minutes. The total distance traveled by each mouse was recorded *via* VisuTrack 3.0 software (Shanghai Xin Luan MDT Infotech Ltd., China).

NOR tests: The same square plastic box used in the OFTs was also used in this study. The NOR test consists of a training phase and a testing phase. In the training phase, two identical objects were positioned in a corner 5 cm away from each adjacent arena wall. The mice were gently placed in the chamber with their back to the objects and allowed to explore freely for 10 min. NOR testing was performed after the mice had rested for twenty-four hours. One of the two identical objects was replaced with a completely different object. The mice were then returned to the arena in the same position for another 5 min of exploration. The time spent exploring the old and novel objects was recorded *via* a video-tracking system. Exploration was defined as approaching an object within 1.5 cm or sniffing or touching an object with the forepaws, vibrissae or snout. The recognition index was calculated as [(exploration time for the novel object)/(total exploration time for both objects) × 100%] to investigate the memory retention of each mouse.

Y maze tests: A Y-shaped maze with three opaque arms (56 × 16 × 32 cm, RWD Life Science, Shenzhen, China) at a 120° angle from each other was used to test the spontaneous alteration rate to assess learning and spatial memory. Briefly, the mice were gently placed individually in the center of the Y maze for 8 min to allow free exploration. The number and sequence of arm entries were recorded by a video-tracking system. A spontaneous alternation was defined as a mouse entering all three arms consecutively without repeats, such as ABC, ACB, BAC, BCA, CAB or CBA. The spontaneous alteration rate was calculated as [(number of spontaneous alterations)/(total arm entries - 2)] × 100%.

### 2.4 Effects of Lingo1 overexpression in hippocampal neurons on cognitive function

Eight-week-old mice were used to evaluate the effects of Lingo1 overexpression in hippocampal neurons on cognitive function. In brief, the mice were mounted in a stereotaxic instrument (RWD Life Science Co., Ltd., Shenzhen, China) after deep anesthesia with tribromoethanol. An adeno-associated virus expressing full-length *Lingo1* (AAV2/9-hSyn-Lingo1-3xFLAG-P2A-EGFP-WOPE, named Lingo1, 5.0 × 10^12^ v.g./mL, OBiO Technology (Shanghai) Co., Ltd., China) was stereotaxically injected into the bilateral hippocampal CA1 and CA3 subregions (coordinates of CA1: -2.1 mm anteroposterior from bregma; ±1.9 mm mediolateral; -1.6 mm dorsoventral from the dura; coordinates of CA3: -2.1 mm anteroposterior from bregma; ±2.3 mm mediolateral; -2.1 mm dorsoventral from the dura) *via* a Hamilton Neuros syringe 1701 KH (Romania) at a rate of 0.2 μL/min, with a volume of 1.5 μL per site. The needle was allowed to leave in place for at least 5 min after each injection. During this procedure, the mice were placed on a homeothermic heating pad to maintain body temperature. Equal volumes of PBS (named vehicle) or empty virus encoding EGFP (AAV2/9-hSyn-3xFLAG-P2A-EGFP-WOPE, named EGFP, 5.0 × 10^12^ v.g./mL) were injected into the same sites as the blank control and negative control, respectively. The constructs were allowed to be expressed for four weeks before the mice were subjected to behavioral tests.

### 2.5 Effects of Lingo1 knockdown in hippocampal neurons on cognitive deficits after anesthesia and surgery

Fifteen-month-old mice were used to investigate the effects of Lingo1 knockdown in hippocampal neurons on the occurrence and development of POCD. The adeno-associated virus AAV2/9-U6-shRNA(Lingo1)-CMV-EGFP-WPRE (named shRNA(Lingo1), 2.0 × 10^12^ v.g./mL, OBiO Technology (Shanghai) Co., Ltd., China) or AAV2/9-U6-shRNA(NC2)-CMV-EGFP-WPRE (2.93 × 10^12^ v.g./mL) was injected into the bilateral hippocampal CA1 and CA3 subregions through the procedure described above. Three weeks after virus injection, all the mice underwent unilateral nephrectomy as described above. Thereafter, neurobehavioral tests were carried out to examine cognitive function on day 4 after surgery.

### 2.6 Transmission electron microscopy (TEM)

Randomly selected mice from each group were deeply anesthetized and perfused through the heart with a mixture of 4% paraformaldehyde and 3% glutaraldehyde. The brains were then excised and postfixed in 1% osmium tetroxide. Afterward, the brains were infiltrated and embedded in Epon812 after dehydration in a series of acetone solutions. Ultrathin sections (60~90 nm) were obtained on an ultramicrotome (Leica, Germany) and then stained with uranyl acetate and lead citrate. The myelin sheaths were observed and detected with a JEM-1400-FLASH transmission electron microscope (JEOL, Japan). The extent of axonal myelination was evaluated by calculating the *G*-ratio *via* the following formula: *G*-ratio = axon diameter of myelinated nerve fibers/outer myelin diameter of myelinated nerve fibers. ImageJ 1.54f software (National Institutes of Health, USA) was used for measurement of the axonal caliber and axonal counting.

### 2.7 Electrophysiology

Artificial cerebrospinal fluid (ACSF) was prepared by dissolving 185 mM sucrose, 20 mM D-glucose, 2.5 mM KCl, 1.25 mM NaH_2_PO_4_, 26 mM NaHCO_3_, 1 mM CaCl_2_ and 6 mM MgCl_2_ in sterile deionized water at a pH of 7.4 and saturated with 95% O_2_ and 5% CO_2_ before use. Then, whole brains from the euthanized mice were quickly removed and transferred to ice-cold ACSF. The brains were subsequently cut into slices at a thickness of 350 mm on a vibrating microtome (Leica Microsystems). Hippocampal cross sections were recovered at 32℃ for 30 min in ACSF containing 124 mM NaCl but not sucrose. After preincubation for at least 60 min at room temperature, the slices were transferred into a glass bottom chamber and completely immersed in new ACSF with continuous bubbling at 32℃ constant temperature. The new ACSF was prepared by dissolving 124 mM NaCl, 20 mM glucose, 2.5 mM KCl, 1.25 mM NaH_2_PO_4_, 26 mM NaHCO_3_ and 4 mM CaCl_2_ in deionized water at pH 7.4.

A glass pipette electrode was used for field recordings under current clamp mode after being filled with ACSF. A bipolar metal stimulating electrode was used to evoke field excitatory postsynaptic potentials (fEPSPs). Afterward, long-term potential (LTP) was induced by a θ-burst, which included six episodes with ten second intervals. Each episode of a θ-burst contained five 5 Hz bursts, and each burst contained five 100 Hz pluses. fEPSPs were recorded for 15 min at baseline and 50 min after induction at a frequency of 0.1 Hz. LTP was calculated by averaging the normalized fEPSP slope values 40~50 min after high-frequency stimulation. An Axon Patch 700B amplifier was used to record data with 10 kHz sampling and 2 kHz low-pass filtering. All the data were analyzed offline on Clampfit 10 (Molecular Devices).

### 2.8 Statistical analysis

All analyses were performed via GraphPad Prism 8.0.2 (San Diego, CA). The quantitative data are shown in bar graphs as the means ± standard deviations. Two-tailed unpaired Student's t tests were used to analyze the significance between two samples, and one-way analysis of variance (ANOVA) with Tukey's post- hoc test was used for multiple comparisons. All experiments were carried out at least in triplicate, and *P* < 0.05 was considered to indicate statistical significance.

## 3. Results

### 3.1 Unilateral nephrectomy led to cognitive deficits in aged mice

**Figure 1:**
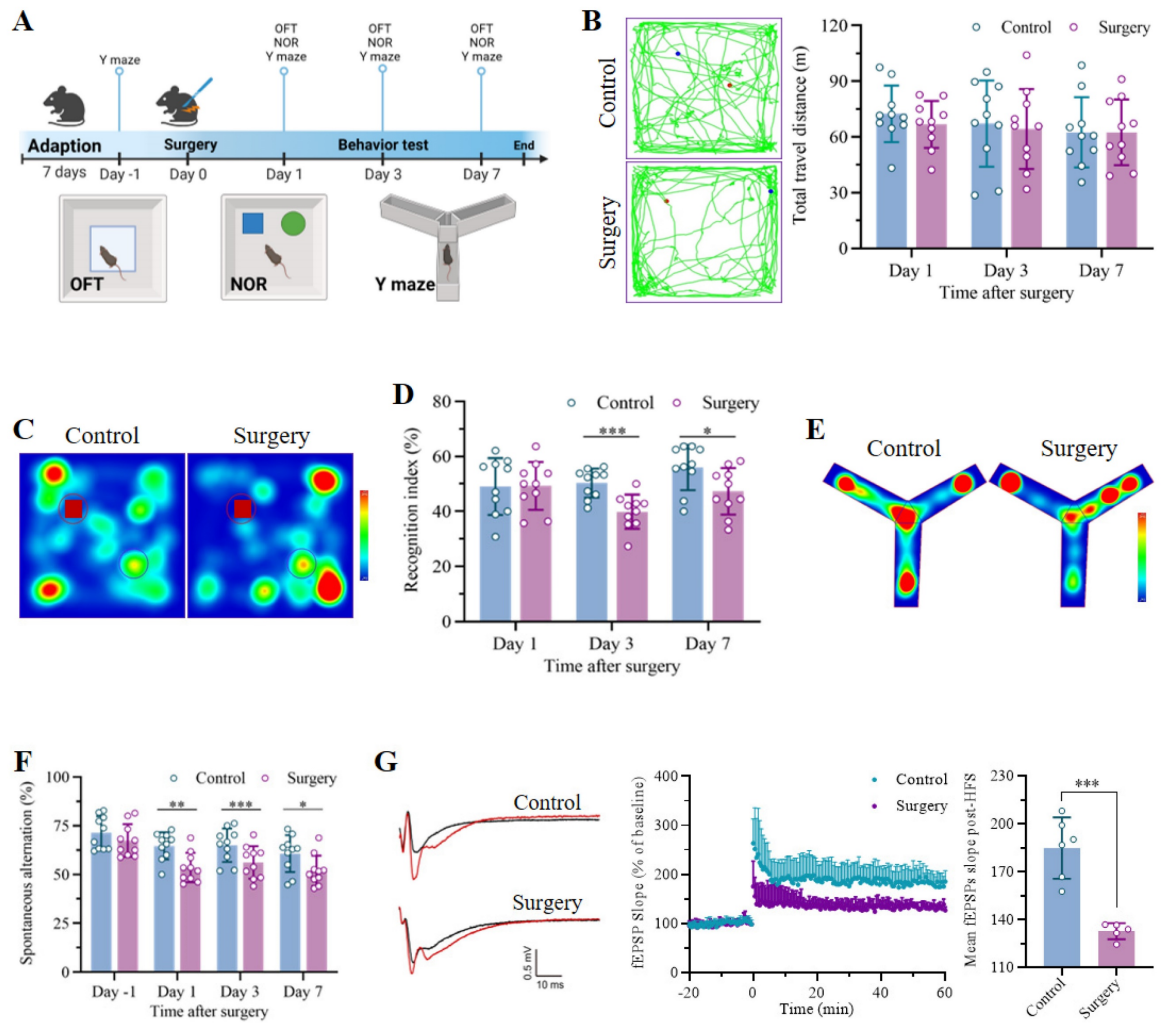
Unilateral nephrectomy under isoflurane anesthesia induced significant cognitive deficits in aged mice. A. Flowchart showing the timeline of the experimental procedures. Fifteen-month-old mice were subjected to unilateral nephrectomy under anesthesia after at least 7 days of adaptation. The open field test (OFT), novel object recognition test (NOR) and Y maze were subsequently conducted on days 1, 3 and 7 after surgery to assess cognitive function. Age-matched mice without any treatment were used as controls. B. Representative trajectories in the OFTs were recorded on day 3 after surgery, and the total travel distance was measured on days 1, 3 and 7 after surgery (n = 10). C. Representative trajectory heatmaps from the NOR test were generated on postoperative day 3. D. Recognition indices in NOR tests were examined on days 1, 3 and 7 after surgery (n = 10). E. Representative trajectory heatmaps of the Y maze test results were generated on day 3 after surgery. F. The spontaneous alternations of each group in the Y maze test were analyzed to assess anesthesia and surgery-induced cognitive decline (n = 10). G. Representative traces of fEPSPs before and after high-frequency stimulation (HFS, left). The effects of anesthesia surgery on LTP in the hippocampus were examined by recording fEPSPs (middle). Cumulative data showing the measurement of mean fEPSPs post-HFS (right). All the statistical data are presented as the mean ± standard error. *P < 0.05, **P < 0.01, ***P < 0.001.

The POCD model was established through unilateral nephrectomy under isoflurane anesthesia, as shown in the flowchart in** Figure 1A**. The mice in the control group underwent neither anesthesia nor surgery. No significant differences in the total distance traveled during the 5 min of exploration in the OFT were detected between the control and surgery groups (**Figure 1B**), revealing negligible effects of the surgical procedure on locomotor ability. The NOR and Y maze tests were subsequently carried out to assess the cognitive function of the mice. In the NOR test, the exploration of the familiar objects in the training phase did not differ between the groups, indicating equal baseline cognitive abilities. However, the recognition indices of the mice that received anesthesia and surgery were markedly lower than those of the mice that did not undergo surgery on postoperative days 3 and 7 (**Figure 1C-D and S1A-C**). In the Y maze test, compared with that of the control group, the spontaneous alternation rate of the mice that underwent unilateral nephrectomy was significantly lower at the corresponding time points. Nevertheless, no significant differences in the spontaneous alternation ratio before surgery or in the total number of arm entries were detected between the two groups, indicating a comparable cognitive baseline and a negligible effect of surgery on locomotor activity (**Figure 1E-F and S1D**). Finally, electrophysiology was performed to measure the fEPSP slope, and the results demonstrated that LTP of the hippocampus significantly decreased in the mice that underwent surgery under anesthesia (**Figure 1G**). In summary, these results indicated that unilateral nephrectomy under isoflurane anesthesia had no effect on motor function but led to remarkable cognitive dysfunction in a time-dependent manner.

### 3.2 Surgery under anesthesia upregulated Lingo1 in the hippocampal neurons of aged mice

**Figure 2:**
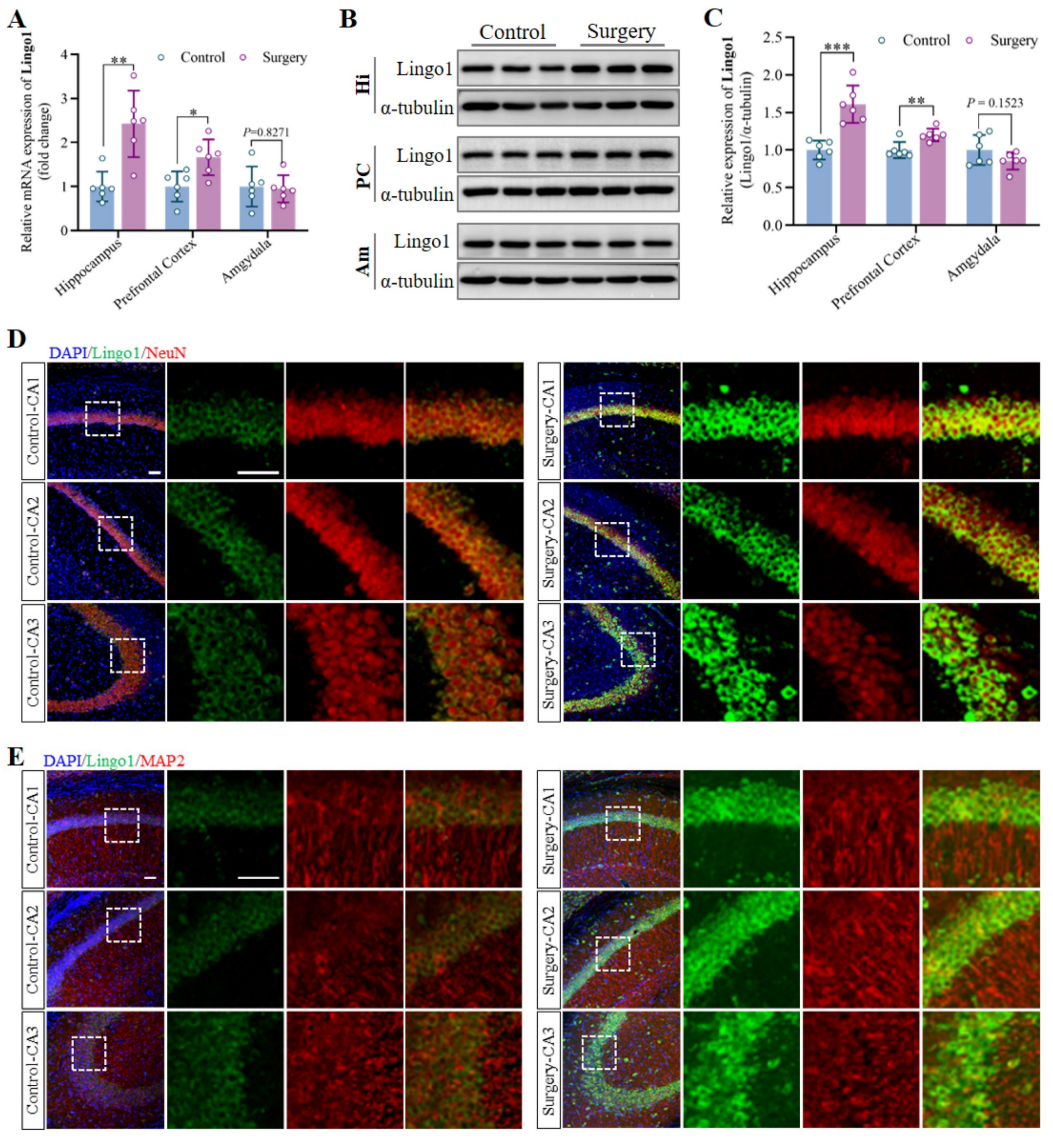
The upregulation of Lingo1 in hippocampal neurons might be associated with the occurrence and development of cognitive decline induced by anesthesia surgery. A. Expression of *Lingo1* mRNA in the hippocampus, prefrontal cortex and amygdala was measured by qRT-PCR (n = 6). B. Representative western blots of Lingo1 in the hippocampus, prefrontal cortex and amygdala on postoperative day 3. C. Expression of the Lingo1 protein in different brain regions at 3 days post-surgery was determined *via* western blot analysis (n = 6). D-E. Representative confocal images of Lingo1 expression in neurons (D: NeuN; E: MAP2) in the hippocampal CA1, CA2 and CA3 regions. Blue: DAPI; Green: Lingo1; Red: NeuN and MAP2. Scale bar: 10 μm. All the statistical data are presented as the mean ± standard error. *P < 0.05, **P < 0.01, ***P < 0.001.

The hippocampus, prefrontal cortex and amygdala were collected on day 3 after anesthesia and surgery, and Lingo1 expression at the mRNA and protein levels was measured through qRT-PCR and western blot analysis. The results revealed that the mRNA and protein expression of Lingo1 in the hippocampus and prefrontal cortex in the surgery groups was markedly greater than that in the control group. Nevertheless, no significant differences in Lingo1 expression at either the mRNA or protein level were detected in the amygdala at 3 days post-surgery (**Figure 2A-C**). Notably, Lingo1 expression in the hippocampus after surgery was much greater than that in the prefrontal cortex. Therefore, dual immunofluorescence staining was performed by combining Lingo1 with markers for neurons (MAP2 and NeuN), astrocytes (GFAP), microglia (Iba1) and oligodendrocytes (OLIG2) to assess Lingo1 expression in different hippocampal subregions and neural cells. The results demonstrated that the bright green fluorescent signals of Lingo1 were localized mainly in neurons in the hippocampal CA1, CA2 and CA3 regions rather than in other neural cells (**Figure 2D-E and S2**). In general, these results suggest that the upregulation of Lingo1 in hippocampal neurons might be closely related to the occurrence and development of surgery-induced cognitive deficits.

### 3.3 Upregulation of Lingo1 in hippocampal neurons led to cognitive deficits after surgery under anesthesia

**Figure 3:**
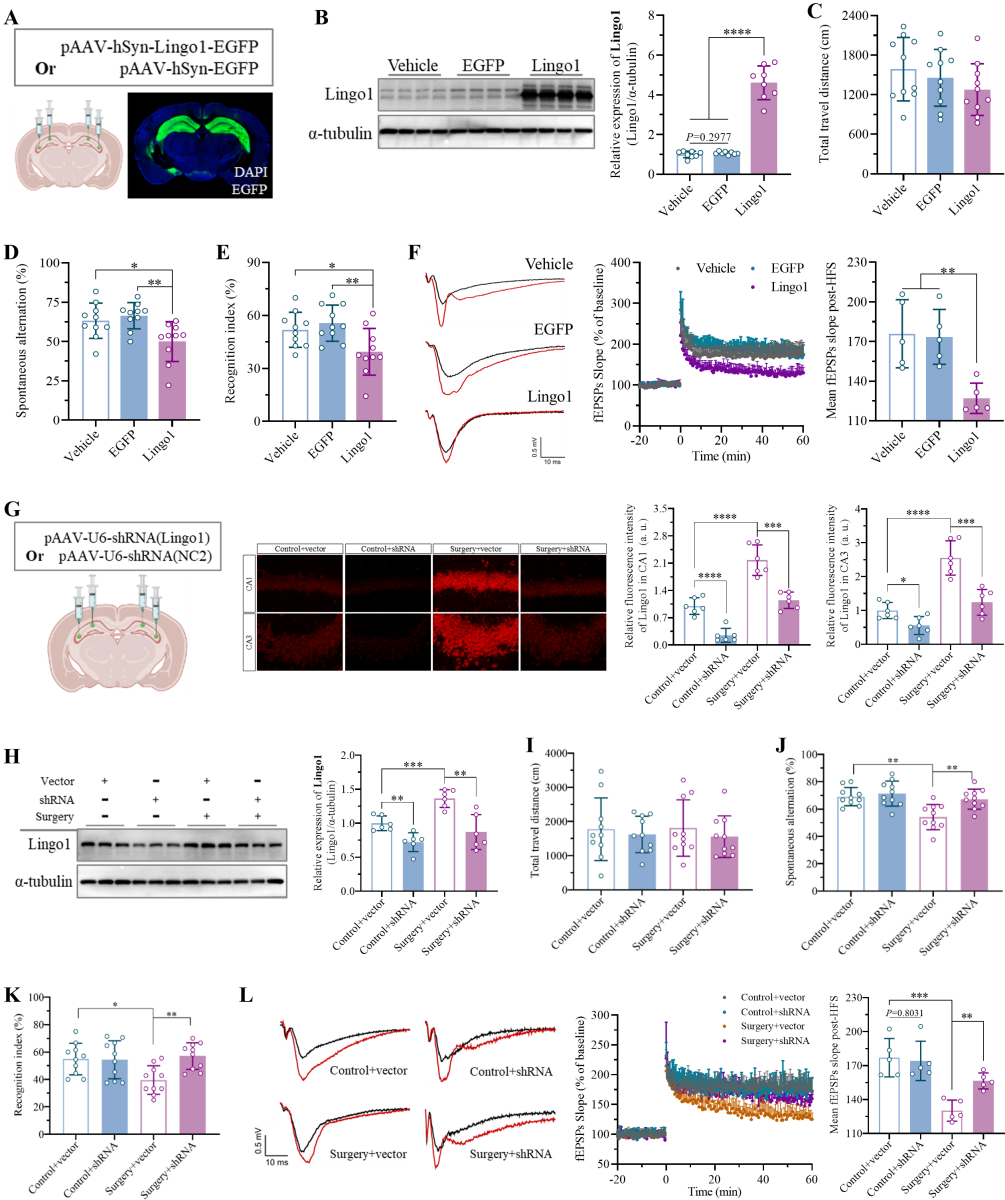
Upregulation of Lingo1 in hippocampal neurons led to cognitive deficits after anesthesia surgery. A. Schematic representation of virus injection and a representative confocal image of Lingo1-EGFP in the hippocampus. B. Lingo1 expression in the hippocampus was determined by western blot analysis on day 28 after Lingo1 transfection (n = 8). C-E. Cognitive function and locomotor activity were evaluated by detecting total travel distance in OFT tests (C, n = 10), spontaneous alterations in Y maze experiments (D, n = 10) and cognitive indices in NOR tests after Lingo1 transfection for 28 days (E, n = 10). F. Representative traces of fEPSPs before and after HFS (left). Effects of Lingo1 upregulation on LTP in the hippocampus was examined by recording fEPSPs (middle). Cumulative data showing the measurement of mean fEPSPs post-HFS (right). G. Schematic representation of virus injection and representative confocal images and quantification of Lingo1 expression in the hippocampal CA1 and CA3 subregion (n = 6). H. Lingo1 expression in the hippocampus was detected through western blot analysis after Lingo1 was knocked down in the hippocampus, followed by anesthesia and surgery (n = 6). I-K. After Lingo1 expression in hippocampal neurons was successfully knocked down *via* shRNA injection, cognitive function and locomotor activity were evaluated by recording total travel distance in OFT tests (I, n = 10), spontaneous alterations in Y maze experiments (J, n = 10) and recognition indices in NOR tests (K, n = 10). L. Representative traces of fEPSPs before and after HFS (left). Effects of Lingo1 knockdown on surgery-induced LTP impairment in the hippocampus were examined by recording fEPSPs (middle). Cumulative data showing the measurement of mean fEPSPs post-HFS (right). All the statistical data are presented as the mean ± standard error. *P < 0.05, **P < 0.01, ***P < 0.001, ****P < 0.0001.

To determine the role of Lingo1 in the development of surgery-induced cognitive decline, we artificially upregulated the expression of Lingo1 in hippocampal neurons *via* stereotaxic microinjection of an adeno-associated virus encoding the full-length mouse *Lingo1* gene. Equal volumes of vehicle or empty adeno-associated vector were injected into the same sites as controls. Confocal images and western blotting revealed that Lingo1 expression was successfully upregulated in hippocampal neurons 28 days after transfection (**Figure 3A-B**). The effects of Lingo1 overexpression in hippocampal neurons on cognitive function were subsequently examined through neurobehavioral tests. The OFT results revealed no obvious differences in total travel distance among the vehicle, EGFP and Lingo1 groups (**Figure 3C and S3A**). In the Y maze test, the spontaneous alteration rate was significantly lower in the Lingo1-overexpressing group than in the control group, but there were no differences between the vehicle and EGFP groups (**Figure 3D**). Moreover, the total number of arm entries was not markedly different among these three groups (**Figure S3B**). In the NOR test, the exploration of the three familiar objects in the training phase did not differ among the groups (**Figure S3C**), although the recognition index in the Lingo1-overexpressing group decreased significantly (**Figure 3E**). Interestingly, the LTP slope in hippocampal neurons was markedly lower in Lingo1-overexpressing mice than in control mice (**Figure 3F**). These results revealed that the abnormal upregulation of Lingo1 in hippocampal neurons is closely related to postoperative cognitive deficits.

Furthermore, Lingo1 expression in hippocampal neurons was knocked down to pinpoint its potential role in surgery-induced cognitive impairment in aged mice. As shown in **Figure 3G**, a serotype 2/9 adeno-associated virus harboring a shRNA sequence against *Lingo1* was generated and stereotactically injected into the bilateral hippocampal CA1 and CA3 subregions. Next, the mice underwent unilateral nephrectomy on day 21 post-injection, and Lingo1 expression in the hippocampal neurons was examined* via* confocal microscopy and western blotting 3 days after surgery. Under microscopy, the dim fluorescence signals were localized in neurons, demonstrating the effective inhibition of Lingo1 expression, which was further confirmed through western blotting (**Figure 3G-H**). We subsequently examined the influence of Lingo1 knockdown in hippocampal neurons on cognitive function after surgery in aged mice through the OFTs, Y maze test and NOR test. In the OFT tests, the negligible difference in the total travel distance among the groups indicated slight influences of virus injection and surgery on the locomotor function of the aged mice (**Figure 3I and S4A**). The nonsignificant differences in total arm entries in the Y maze test further suggested that virus injection and unilateral nephrectomy did not markedly influence locomotor activity (**Figure S4B**). However, the spontaneous alternation rate did not differ between the control mice injected with the empty vector and those injected with shRNA(Lingo1), revealing that adeno-associated virus injection into the hippocampus did not affect cognitive function. The spontaneous alternation rate of the mice treated with the empty vector substantially decreased after unilateral nephrectomy, which was dramatically attenuated by the stereotactic injection of shRNA(Lingo1) into the hippocampus (**Figure 3J and S4B**). In the NOR test, no differences in the recognition index were observed between the mice injected with only the AAV vector or shRNA(Lingo1). Predictably, unilateral nephrectomy markedly reduced the recognition index in aged mice injected with empty vector. When Lingo1 expression in hippocampal neurons was knocked down by shRNA(Lingo1), cognitive impairment was substantially attenuated (**Figure 3K and S4C**). We further found that LTP in the hippocampus was markedly lower in the mice in the surgery group than in the control groups by calculating the fEPSP slope. When the expression of Lingo1 in hippocampal neurons was knocked down by shRNA(Lingo1), the LTP slope was markedly elevated (**Figure 3L**). Taken together, these data demonstrated that abnormal Lingo1 upregulation in hippocampal neurons plays a crucial role in the occurrence and development of surgery-induced cognitive impairment.

### 3.4 Lingo1 upregulation in hippocampal neurons led to cognitive decline after surgery *via* myelin sheath loss

**Figure 4:**
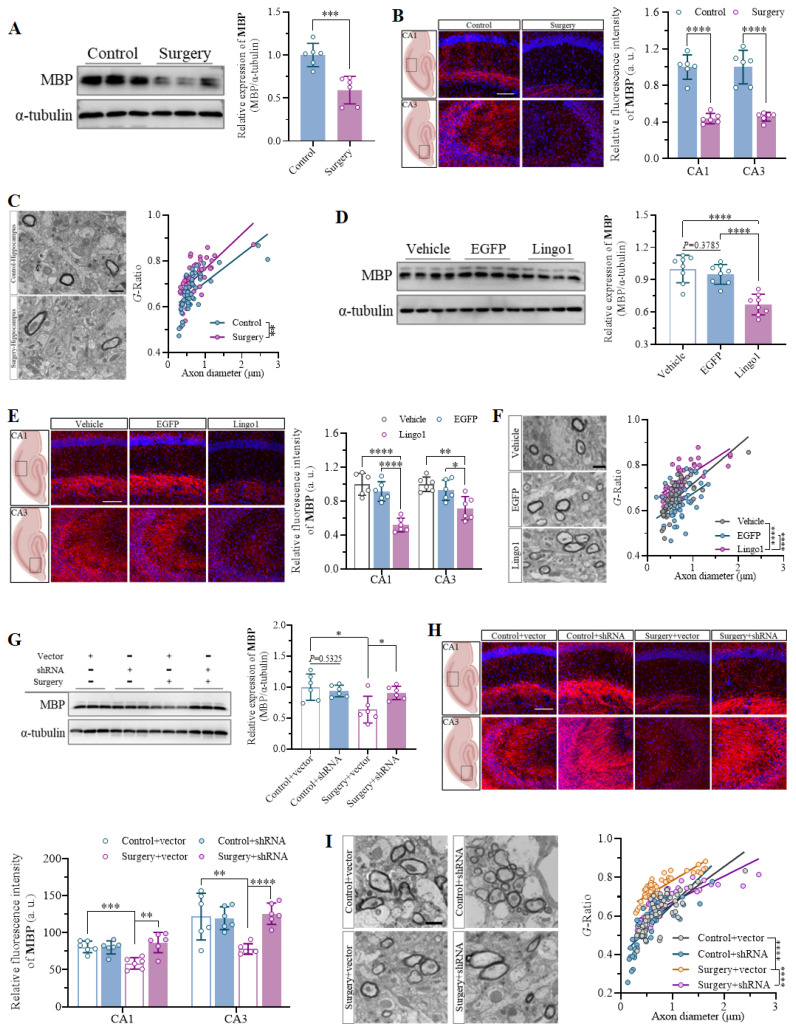
Upregulation of Lingo1 in hippocampal neurons mediated cognitive decline post-operation by promoting myelin sheath loss. A. Representative immunoblot bands and quantification of MBP in the hippocampus of aged mice after surgery (n = 6). B. Representative confocal images and quantification of MBP in the hippocampal CA1 and CA3 regions in the control and surgery groups (n = 6). Scale bar: 20 μm. C. Representative TEM images of the hippocampus and scatterplots of the myelin *G*-ratios in aged mice after surgery. D. Representative immunoblot bands and quantification of MBP in the hippocampi of vehicle-, EGFP- and Lingo1-transfected mice (n = 6). E. Representative immunofluorescence images and quantification of MBP in the hippocampal CA1 and CA3 regions in vehicle-, EGFP- and Lingo1-injected mice (n = 6). Scale bar: 20 μm. F. Representative TEM images and quantification of myelin sheath thickness in the hippocampus of vehicle-, EGFP- and Lingo1-injected mice. G. Representative western blot images and quantification of MBP in the hippocampus of mice subjected to Lingo1 knockdown and anesthesia surgery (n = 6). H. Representative confocal images and quantification of MBP in the hippocampal CA1 and CA3 subfields of the hippocampus of the mice underwent Lingo1 knockdown and anesthesia surgery (n = 6). Scale bar: 20 μm. I. Representative TEM images and quantification of myelin sheath thickness in the hippocampus of mice underwent Lingo1 knockdown and anesthesia surgery. All the statistical data are presented as the mean ± standard error. *P < 0.05, **P < 0.01, ***P < 0.001, ****P < 0.0001.

Myelin formation and good structural integrity are crucial foundations for the function of neuronal axons in the central nervous system^26^. Therefore, the overall levels of MBP expression in the hippocampus were first quantified through western blot analysis and immunostaining to examine whether anesthesia and surgery led to considerable changes in myelination. The results indicated that unilateral nephrectomy under anesthesia markedly decreased MBP expression in the hippocampus of aged mice (**Figure 4A and B**). Furthermore, abnormalities in the submicroscopic structure of myelin sheaths and an increased *G*-ratio were observed in the hippocampus through TEM, which demonstrated significant impairment of myelin sheaths in aged mice after surgery (**Figure 4C**). Given that Lingo1 is a vital negative regulator of myelination^20^, MBP expression and myelin sheaths in the hippocampus were investigated in mice with different levels of Lingo1 expression in hippocampal neurons. As shown by the western blot and immunofluorescence results, the expression of MBP was dramatically lower in the hippocampi of the mice with Lingo1 overexpression than in those of the control mice, suggesting that Lingo1 substantially promoted myelin loss in the hippocampus (**Figure 4D and E**). The increase in the *G*-ratio in Lingo1-overexpressing mice further revealed that the upregulation of Lingo1 in hippocampal neurons led to thinning of the myelin sheath (**Figure 4F**). To further investigate whether myelin loss is associated with the upregulation of Lingo1 induced by anesthesia and surgery, Lingo1 expression in hippocampal neurons was knocked down through an shRNA(Lingo1) before surgery. The results revealed significant myelin sheath injury in the mice subjected to surgery and injected with empty virus, as indicated by decreased MBP expression and an increased *G*-ratio. When Lingo1 expression was knocked down through shRNA(Lingo1) injection, the downregulation of MBP expression and increase in the *G*-ratio caused by surgery were reversed (**Figure 4G-I**). These results further revealed that the upregulation of Lingo1 in hippocampal neurons might be a key factor in regulating myelin sheath impairment, which plays crucial roles in the development of the cognitive impairment caused by surgery.

### 3.5 Lingo1 upregulation in hippocampal neurons led to cognitive dysfunction after surgery *via* abnormal tau phosphorylation

**Figure 5:**
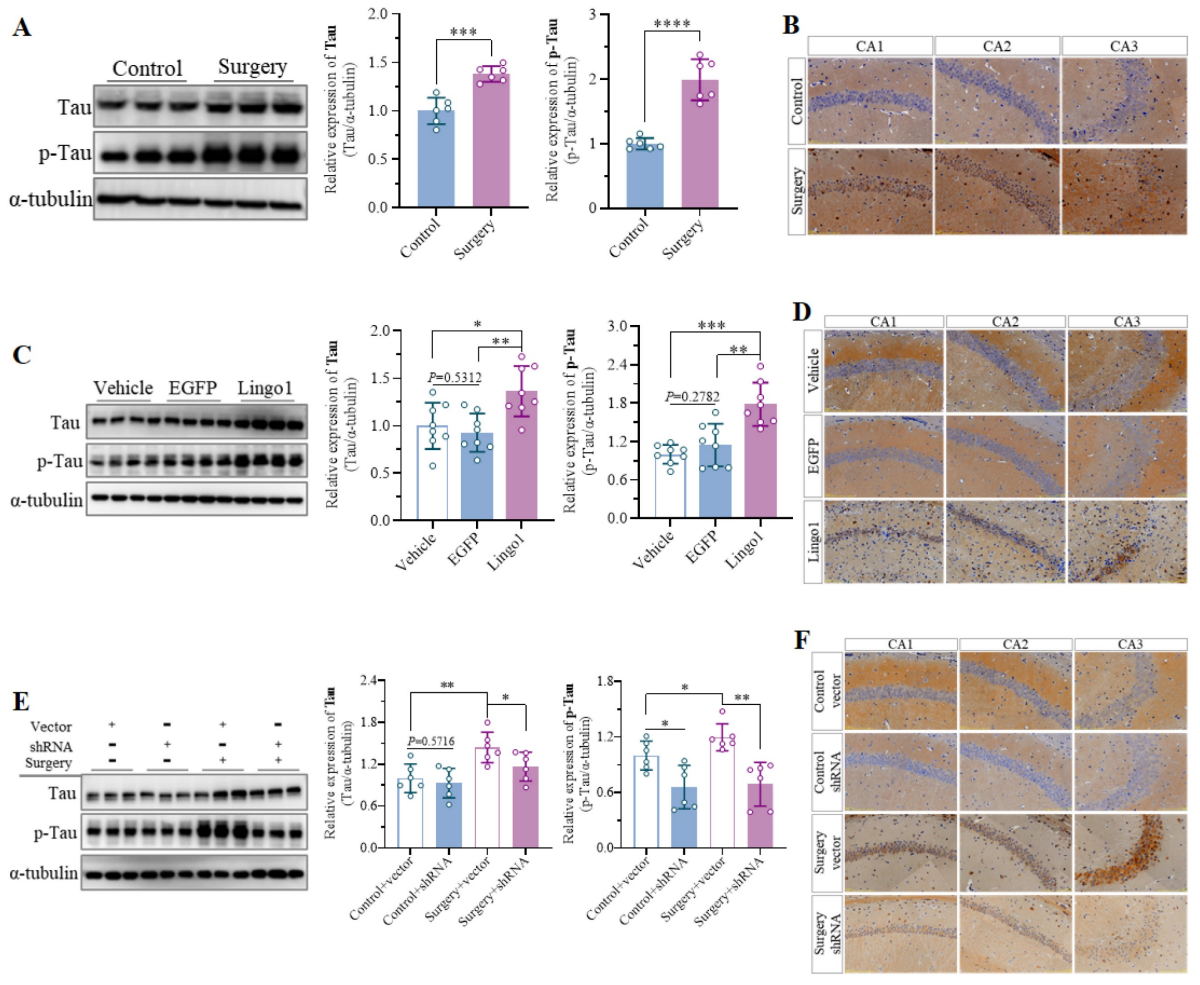
Lingo1 upregulation in hippocampal neurons is involved in POCD by promoting abnormal tau protein phosphorylation. A. Representative immunoblot bands and quantification of tau and phosphorylated tau protein in the hippocampus of aged mice after anesthesia and surgery (n = 6). B. Immunohistochemistry showed increased levels of abnormally phosphorylated tau protein in the hippocampus. C. Representative immunoblot bands and quantification of tau and phosphorylated tau protein in the hippocampus of vector-, EGFP- and Lingo1-injected mice (n = 8). D. Lingo1 overexpression in hippocampal neurons significantly promoted abnormal tau phosphorylation, as detected by immunohistochemistry. E. Representative immunoblots and quantification of tau and phosphorylated tau protein in the hippocampus of mice underwent Lingo1 knockdown and anesthesia surgery (n = 6). F. Immunohistochemistry results demonstrated that the knockdown of Lingo1 in hippocampal neurons inhibited anesthesia surgery-induced abnormal phosphorylation of the tau protein. All the statistical data are presented as the mean ± standard error. *P < 0.05, **P < 0.01, ***P < 0.001, ****P < 0.0001.

Increasing evidence has indicated that abnormal phosphorylation of the tau protein plays important roles in the pathogenesis of cognitive dysfunction caused by surgery under anesthesia^32^. Therefore, tau protein expression and phosphorylation in the hippocampus on day 3 after surgery were first detected through western blotting and immunohistochemistry. Tau protein expression and abnormal tau phosphorylation in the hippocampus were elevated in mice subjected to the surgical procedure compared with those in control mice at the same time points (**Figure 5A-B**). Moreover, the expression and phosphorylation of the tau protein were further measured in mice with different Lingo1 expression levels to determine whether the dysregulation of Lingo1 in hippocampal neurons is involved in abnormal tau phosphorylation. Interestingly, the levels of phosphorylated tau protein in the hippocampus were markedly greater in the mice with Lingo1 overexpression than in the mice injected with vehicle or EGFP, but tau expression was not markedly different (**Figure 5C-D**). Additionally, the increase in the level of the tau protein and abnormal tan phosphorylation after surgery were ameliorated when the Lingo1 protein was knocked down by shRNA(Lingo1) (**Figure 5E-F**). These data suggest that the anomalous upregulation of Lingo1 in hippocampal neurons after unilateral nephrectomy might lead to cognitive decline by promoting abnormal tau phosphorylation.

### 3.6 Surgery-induced Lingo1 upregulation in hippocampal neurons activated the RhoA/ROCK1 pathway through interaction with p75NTR/NgR

**Figure 6:**
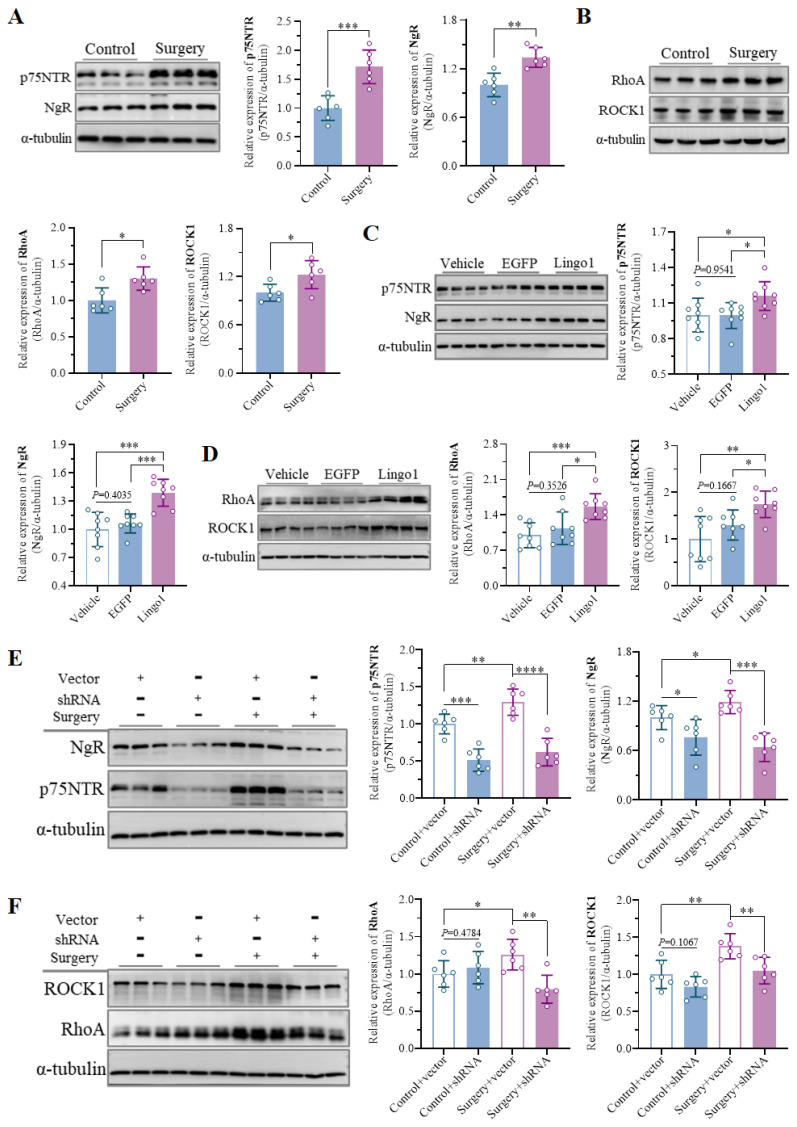
Lingo1 upregulation in hippocampal neurons activated the RhoA/ROCK1 pathway by cooperating with p75NTR and NgR in the development of POCD. A-B. Representative immunoblot bands and quantification of (A) p75NTR/NgR and (B) RhoA/ROCK1 in the hippocampus of aged mice after anesthesia surgery (n = 6). C-D. Representative immunoblot bands and quantification of (C) p75NTR/NgR and (D) RhoA/ROCK in the hippocampus after Lingo1 transfection for 28 days (n = 8). E-F. Representative immunoblots and quantification of (E) p75NTR/NgR and (F) RhoA/ROCK1 in the hippocampus of mice subjected to Lingo1 knockdown and anesthesia surgery (n = 6). All the statistical data are presented as the mean ± standard error. *P < 0.05, **P < 0.01, ***P < 0.0001, ****P < 0.0001.

Owing to its prominent role in myelination and myelin-related processes, Lingo1 has been widely studied in the pathology of neurological conditions, such as multiple sclerosis and spinal cord injury. However, the underlying mechanisms by which Lingo1 regulates cognitive decline after surgery remain to be further elucidated. Previous studies have shown that Lingo1 generally mediates the activity of myelin inhibitors by interacting with both NgR and p75NTR^19^, ^21^. Interestingly, compared with the control treatment, unilateral nephrectomy under isoflurane anesthesia significantly increased NgR and p75NTR expression in the hippocampus (**Figure 6A**). Moreover, the expression of NgR and p75NTR was also increased in the mice with Lingo1 overexpression, but no significant differences were detected between the mice treated with vehicle and those treated with EGFP (**Figure 6C**). When Lingo1 expression was knocked down through shRNA transfection, the expression of NgR and p75NTR in the hippocampus was markedly inhibited (**Figure 6E**).

The RhoA/ROCK signaling pathway plays crucial roles in many central nervous system disorders as an important downstream pathway of Lingo1. We further examined the expression of RhoA and ROCK1 in aged mice after surgery, which demonstrated significant upregulation of RhoA and ROCK1 in the hippocampus (**Figure 6B**). The significant upregulation of RhoA/ROCK1 in the mice with Lingo1 overexpression further indicated that RhoA/ROCK1 activation was mediated by the upregulation of Lingo1 in the hippocampal neurons (**Figure 6D**). Intriguingly, the expression of RhoA and ROCK1 was also increased after the empty vector-treated mice underwent anesthesia and surgery, and this increase was dramatically reversed when Lingo1 was knocked out by shRNA (**Figure 6F**). These results indicate that the upregulation of Lingo1 in hippocampal neurons is a key factor in activating the RhoA/ROCK1 signaling pathway, which is generally involved in this process through cooperation with p75NTR and NgR.

### 3.7 Lingo1 is involved in surgery-induced cognitive impairment by inhibiting the EGFR/PI3K/Akt pathway

Numerous studies have shown that endogenous Lingo1 plays critical roles in negatively regulating the EGFR/PI3K/Akt signaling pathway in the pathophysiology of central nervous system disorders by reducing EGFR levels through direct physical interactions^19^.

**Figure 7:**
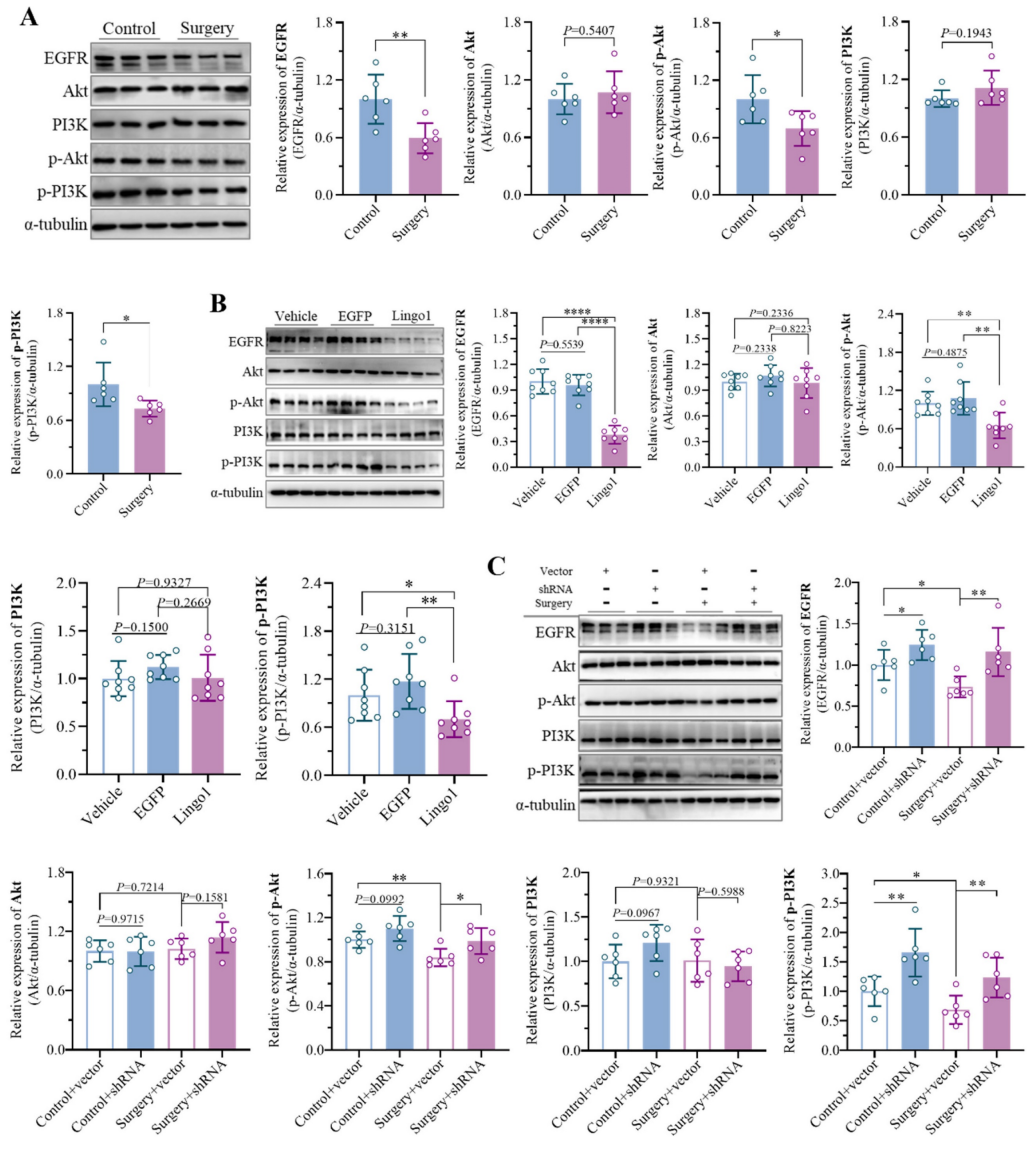
Lingo1 upregulation in hippocampal neurons promoted cognitive decline after anesthesia and surgery by inhibiting the EGFR/PI3K/Akt signaling pathway. A. Representative immunoblot bands and quantification of EGFR, PI3K, Akt, phosphorylated PI3K (p-PI3K) and phosphorylated Akt (p-Akt) in the hippocampus of aged mice after anesthesia and surgery (n = 6). B. Representative immunoblot bands and quantification of EGFR, PI3K, Akt, p-PI3K and p-Akt in the hippocampus 28 days after Lingo1 transfection (n = 8). C. Representative western blotting images and quantification of EGFR, PI3K, Akt, p-PI3K and p-Akt in the hippocampus of the mice with Lingo1 knockdown that underwent surgery (n = 6). All the statistical data are presented as the mean ± standard error. *P < 0.05, **P < 0.01, ****P < 0.0001.

Interestingly, the expression of EGFR was markedly decreased in the mice that underwent unilateral nephrectomy. In addition, the levels of phosphorylated PI3K and Akt were significantly lower in the surgery group than in the control group, while the expression of PI3K and Akt was not significantly different (**Figure 7A**). The results revealed substantial inhibition of the EGFR/PI3K/Akt signaling pathway in the context of the cognitive decline caused by surgery. To provide evidence that surgery-induced Lingo1 upregulation in hippocampal neurons is indispensable for inhibiting the EGFR/PI3K/Akt signaling pathway, the related proteins were further detected in mice with different Lingo1 expression. The levels of phosphorylated PI3K/Akt and EGFR expression were dramatically lower in Lingo1-overexpressing mice than in vehicle- and EGFP-injected mice (**Figure 7B**). Notably, the decreased EGFR and phosphorylated PI3K/Akt levels induced by anesthesia and surgery were substantially reversed through hippocampal neuron-specific Lingo1 knockdown (**Figure 7C**), further demonstrating that the upregulation of Lingo1 in hippocampal neurons plays crucial roles in the development of postoperative cognitive deficits through the inhibition of the EGFR/PI3K/Akt pathway.

### 3.8 Lingo1 upregulation in hippocampal neurons induced neuronal apoptosis

**Figure 8:**
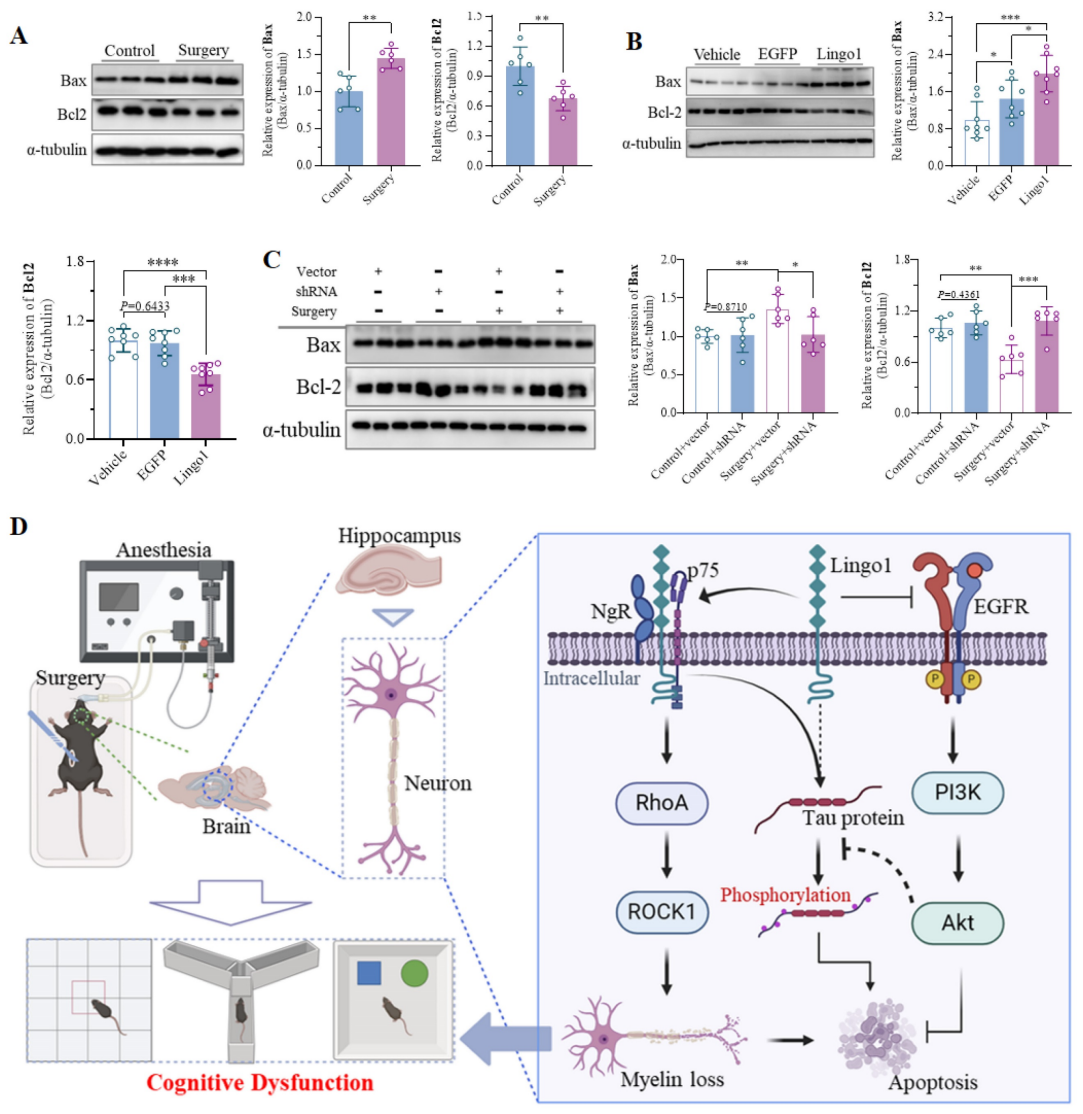
Lingo1 upregulation in hippocampal neurons promoted cognitive decline after anesthesia and surgery by increasing neuronal apoptosis. A. Representative immunoblot bands and quantification of Bax and Bcl2 in the hippocampus of aged mice after anesthesia and surgery (n = 6). B. Representative immunoblot bands and quantification of Bax and Bcl2 in the hippocampi of vehicle-, EGFP- and Lingo1-injected mice (n = 8). C. Representative western blot images of Bax and Bcl2 in the hippocampi of the mice with Lingo1 knockdown and surgery (n = 6). All the statistical data are presented as the mean ± standard error. *P < 0.05, **P < 0.01, ***P < 0.001, ****P < 0.0001. D. General overview of the main highlights of this study.

Bax is a vital proapoptotic factor that regulates cell apoptosis by inducing permeation through the mitochondrial outer membrane^38^. Conversely, Bcl2 plays essential roles in inhibiting oxidative stress, decreasing the secretion of proapoptotic cytokines, regulating the function of apoptotic factors, and maintaining intracellular calcium homeostasis^39^. Notably, as shown in Figure 8A, the expression of Bax in the hippocampus was markedly increased 3 days after unilateral nephrectomy compared with that in control mice, whereas Bcl2 expression was substantially downregulated (**Figure 8A**). Upregulation of Bax and downregulation of Bcl2 were further observed in mice with hippocampal neuron-specific Lingo1 overexpression (**Figure 8B**). Interestingly, the expression of these proteins was dramatically altered when Lingo1 was knocked down by shRNA(Lingo1) (**Figure 8C**). These results demonstrated that the elevation of Lingo1 in the hippocampus also plays a critical role in mediating cognitive decline after surgery by enhancing neural apoptosis. Consequently, we investigated the possible underlying mechanism by which Lingo1 mediates the cognitive dysfunction induced by unilateral nephrectomy under isoflurane anesthesia. As shown in **Figure 8D**, Lingo1 was maintained at physiological levels under normal conditions; however, the expression of Lingo1 was markedly increased in hippocampal neurons after surgery. The upregulated Lingo1 subsequently activated the RhoA/ROCK1 pathway through the formation of a receptor complex with both NgR and p75NTR, which further mediated myelin loss and abnormal tau phosphorylation. Moreover, increased Lingo1 expression in hippocampal neurons promoted neuronal apoptosis by inhibiting the EGFR/PI3K/Akt signaling pathway. These pathological changes caused by Lingo1 upregulation after anesthesia and surgery ultimately result in cognitive impairment.

## Discussion

Accumulating evidence has highlighted the adverse effects of surgery under anesthesia on cognitive function^1^, ^2^. However, the mechanism underlying learning and memory impairment caused by anesthesia and surgery is still not fully understood. In the present study, we established a model of surgery-induced cognitive dysfunction in aged mice through unilateral nephrectomy under isoflurane anesthesia. The results of the behavioral tests demonstrated that the working and recognition memory of the aged mice exhibited significant deficits after surgery but no defects in locomotor activity. Using this mouse model, we found that Lingo1 upregulation in hippocampal neurons is a novel mechanism that mediates cognitive dysfunction after anesthesia and surgery through the combination of molecular biology and animal behavior studies.

Lingo1 is a cell surface glycoprotein encoded by the LRRN6A gene on chromosome 15q24.3r^21^, ^40^. It is specifically expressed in brain tissue and is highly evolutionarily conserved between humans and mice^40^, ^41^. It is usually abnormally upregulated during the pathological progression of neurological disorders, suggesting a deleterious role for the endogenous protein^42^, ^43^. Interestingly, we found significant upregulation of Lingo1 in the hippocampus of aged mice with cognitive impairment after unilateral nephrectomy. Anatomically, the hippocampus proper is composed of CA1, CA2 and CA3 subregions and mainly containing glutamatergic pyramidal cells and GABAergic inhibitory interneurons^44^. Thus, we examined the Lingo1 expression in hippocampal CA1, CA2 and CA3 subregions through immunofluorescence colocalization analysis. The results demonstrated that Lingo1 was upregulated in both excitatory pyramidal neurons and inhibitory interneurons in hippocampus, suggesting that the dysregulation of Lingo1 expression in all hippocampal neurons might ultimately determine the cognitive decline after surgery in aged mice. A previous study demonstrated that Lingo1 expression in the hippocampus was significantly increased in aged rats with spatial learning and memory impairment and was directly proportional to the degree of cognitive decline^23^, ^45^.

In addition, the administration of an anti-Lingo1 antibody markedly ameliorated spatial memory dysfunction in 5xFAD mice by inhibiting the function and expression of Lingo1^46^. These studies strongly indicate that Lingo1 plays substantial roles in the etiology of cognitive impairment and might be a potential therapeutic target. Therefore, we constructed a neuron-specific adeno-associated virus encoding full-length mouse Lingo1 and stereotactically injected them into the bilateral hippocampal CA1 and CA3 subregions as described in previous reports^47^-^49^. Neurobehavioral experiments revealed that mice with hippocampal neuron-specific Lingo1 overexpression exhibited significant cognitive decline. When the expression of Lingo1 was knocked down through the stereotactic injection of an adeno-associated virus expressing a shRNA sequence against Lingo1 into the hippocampus, the cognitive impairment caused by anesthesia surgery was markedly reversed. These results indicate that the abnormal upregulation of Lingo1 in hippocampal neurons is a critical inducer of cognitive impairment after surgery.

The myelin sheaths that wrap axons exhibit optimized electrophysiological signal transduction and provide trophic factors and metabolic substrates to neurons, which is the biological basis of signal transduction in neurons^27^, ^50^. Numerous studies have demonstrated that mature oligodendrocytes and individual myelin sheaths in adult and aging brains typically have good stability; however, new evidence has demonstrated that oligodendrocyte precursor cells continuously differentiate and produce new myelin sheaths throughout life^29^. The formation and structural integrity of myelin are the fundamental requirements for maintaining learning and memory^26^, ^29^. A considerable decrease in myelin generation was observed with increasing age and was associated with cognitive decline^28^, ^51^. Moreover, an increasing number of studies have revealed that impaired myelination and oligodendrocytes are associated with the pathogenesis of numerous psychiatric and neurological disorders, including depression, anxiety, Alzheimer's disease and Parkinson's disease^52^-^55^. Lingo1 is a well-known negative regulator of oligodendrocyte differentiation and axonal myelination^20^. Notably, we observed a significant decrease in myelin levels in the hippocampus of mice that underwent anesthesia and surgery. Decreased myelin levels were also detected in the hippocampus with Lingo1 overexpression. Moreover, myelin loss induced by unilateral nephrectomy under anesthesia was significantly reversed when Lingo1 expression was knocked down by shRNA(Lingo1). These results indicate that the upregulation of Lingo1 in hippocampal neurons plays a substantial role in regulating myelin loss, which is closely related to cognitive decline after surgery. Myelination in the brain is well known to be coordinated by oligodendrocyte differentiation and interactions with axons. Upregulation of Lingo1 in oligodendrocytes has been proven to play a crucial role in inhibiting oligodendrocyte differentiation and axonal myelination^20^. However, abnormal Lingo1 expression was detected in hippocampal neurons but not in oligodendrocytes in this study; moreover, the upregulation of Lingo1 in hippocampal neurons was demonstrated to be closely associated with a reduction in the thickness of myelin sheaths. This phenomenon indicates that the elevated expression of Lingo1 in neurons might also contribute to myelin loss. Interestingly, neurons have been reported to increase oligodendrocyte differentiation or myelin generation in an activity-dependent manner^27^, which further demonstrates that external stimuli-triggered neuronal changes could regulate myelination in an oligodendrocyte-independent manner.

In the past decade of research, myelin-associated glycoproteins, oligodendrocyte-myelin glycoproteins and neurite outgrowth inhibitors have been shown to be important regulators that inhibit neuronal growth and myelination^56^. The mechanism involved in these signaling pathways was initially believed to involve a receptor complex composed of p75NTR and NgR, which inhibits neurite outgrowth and myelination through the RhoA signaling pathway^21^. However, oligodendrocyte-myelin glycoprotein is unable to activate the RhoA pathway in nonneuronal cells coexpressed with p75NTR and NgR, suggesting that additional determinants are required for NgR signal transduction. An important component of the NgR/p75NTR receptor complex, Lingo1, was discovered in previous research^19^, ^21^. Increasing evidence has indicated that Lingo1/NgR/p75NTR plays critical roles in mediating cognitive dysfunction^57^. Interestingly, we detected significant upregulation of NgR and p75NTR in the hippocampus after unilateral nephrectomy, suggesting that Lingo1 potentially interacts with NgR and p75NTR during surgery-induced cognitive impairment. When Lingo1 expression in hippocampal neurons was knocked down by shRNA(Lingo1), the upregulation of NgR and p75NTR was reversed. Moreover, the expression of Lingo1 and p75NTR in the hippocampus was also markedly increased in the mice with Lingo1 overexpression. These results verify that Lingo1 mediates cognitive impairment by interacting with NgR and p75NTR. The RhoA/ROCK signaling pathway is essential for regulating myelination downstream of Lingo1^58^. The expression of RhoA and ROCK1 was significantly upregulated in the hippocampus after surgery under anesthesia, as was the case in mice with Lingo1 overexpression. Moreover, the expression of RhoA and ROCK1 in mice with Lingo1 knockdown was significantly lower than that in mice subjected to unilateral nephrectomy. In summary, Lingo1 upregulation in hippocampal neurons might promote myelination inhibition or myelin sheath loss through the RhoA/ROCK1 pathway, which plays important roles in the occurrence and development of postoperative cognitive decline.

Lingo1 contains a typical EGFR-like tyrosine phosphorylation site in the cytoplasmic domain^59^, ^60^, which can negatively regulate the EGFR/PI3K/Akt pathway through direct binding to EGFR^61^. In the present study, we also determined that Lingo1 upregulation in hippocampal neurons after surgery significantly inhibited the EGFR/PI3K/Akt signaling pathway. Previous studies have indicated that the inhibition of the EGFR downstream signaling pathway might result from the accelerated internalization and degradation of the EGFR protein by Lingo1 on the neuronal membrane. In addition, another study indicated that Lingo1 can reduce neuronal survival and growth by inhibiting the phosphorylation of EGFR and subsequently decreasing the activity of the PI3K/Akt pathway^19^, ^21^. Herein, decreased levels of EGFR were observed in the hippocampus of aged mice that underwent unilateral nephrectomy under anesthesia; therefore, Lingo1 might have accelerated the internalization and degradation of EGFR. EGFR is well known to trigger DNA synthesis and cell proliferation by activating the PI3K/Akt signaling pathway, which is an essential factor in mediating neuronal survival and growth^62^, ^63^. The results of the present study revealed that the expression of proapoptotic protein Bax was significantly elevated, whereas the expression of the antiapoptotic protein Bcl2 was decreased. The expression of these apoptotic factors was further confirmed to be regulated by Lingo1 in hippocampal neurons. Additionally, the abnormal hyperphosphorylation of tau protein is another important factor in the pathogenesis of the cognitive impairment and neuronal apoptosis induced by anesthesia and surgery^64^, ^65^. Previous studies have indicated that p75NTR/NgR and the downstream signaling proteins RhoA/ROCK are regulatory molecules involved in the hyperphosphorylation of the tau protein during AD pathology^36^, ^37^, ^66^. Herein, we also revealed that Lingo1 enhanced abnormal tau phosphorylation in the hippocampus during cognitive decline after anesthesia and surgery, which might also be important for regulating neuronal apoptosis in patients with cognitive deficits induced by anesthesia and surgery^67^.

In summary, our study revealed that abnormal Lingo1 upregulation in hippocampal neurons contributed significantly to cognitive decline after unilateral nephrectomy under isoflurane anesthesia by promoting myelin sheath loss or inhibiting myelination. Elevated Lingo1 expression in hippocampal neurons after surgery was strongly implicated in the activation of the RhoA/ROCK1 signaling pathway through the binding of a receptor complex with p75NTR and NgR. In addition, the expression of this gene increased markedly in response to increased Lingo1 expression in the hippocampus after surgery, which further induced abnormal tau phosphorylation. Moreover, Lingo1 upregulation in hippocampal neurons inhibited the PI3K/Akt signaling pathway by decreasing EGFR levels, which further promoted cognitive decline after anesthesia and surgery by increasing neuronal apoptosis. Importantly, the decreases in myelin levels and neuronal apoptosis in the hippocampus were dramatically inhibited when Lingo1 expression was knocked down by shRNA(Lingo1), which significantly attenuated surgery-induced cognitive impairment. This study provides exciting evidence that Lingo1 could be a promising therapeutic target for the clinical treatment of postoperative cognitive dysfunction.

There are still several limitations in the present work. Herein, we found that Lingo1 expression was significantly increased in the hippocampal neurons of aged mice after unliteral nephrectomy under isoflurane anesthesia and plays essential roles in the development of cognitive decline. The mice that underwent neither surgery nor anesthesia were used as controls, which neglects the effect of isoflurane anesthesia on Lingo1 expression. The lack of a control group treated with only isoflurane anesthesia was a limitation of the experimental design. In addition, on the basis of the same expression patterns of Lingo1, NgR and p75NTR in different groups and previous reports, we concluded that Lingo1 regulates the downstream RhoA/ROCK1 signaling pathway through a receptor complex formed by binding NgR and p75NTR. This conclusion is preliminary and requires further experimental design for validation. Moreover, upregulated Lingo1 in hippocampal neurons contributed to cognitive decline after surgery through activating the RhoA/ROCK1 signaling pathway and inhibiting the EGFR/PI3K/Akt signaling pathway. Nevertheless, the correlations between the RhoA/ROCK1 and EGFR/PI3K/Akt signaling pathways and the key factors involved in the occurrence of surgery-induced cognitive impairment need further experimental verification. Therefore, we will continue to explore the key molecular mechanism by which Lingo1 regulates the development of cognitive deficits after anesthesia and surgery.

## Supplementary Material

Supplementary methods and figures.
