# Supplementary material for: Lingo1 in the hippocampus contributes to cognitive dysfunction after anesthesia and surgery in aged mice
Source: Int J Biol Sci. 2025 Jan 1;21(2):595–613. doi: 10.7150/ijbs.98376 (PMC11705636; doi:10.7150/ijbs.98376)
Supplement: Supplementary file 1 — Supplementary methods and figures. [file ijbsv21p0595s1.pdf]

Supplementary materials for

Lingo1 in the hippocampus contributes to cognitive dysfunction after anesthesia and surgery in aged mice

Changliang Liu[1,2,#], Changteng Zhang[3,4,#], Ling Chen[1,2,5], Xin Liu[1,2], Jiahui Wu[3,4], Yalan Sun[3,4], Jin Liu[3,4,*], Chan Chen[3,4,*]

[1]Department of High Altitude Medicine, and Center for High Altitude Medicine, West China Hospital, Sichuan University, Chengdu, Sichuan 610041, China.

[2]High Altitude Medicine Key Laboratory of Sichuan Province, Institute of High Altitude Medicine, West China Hospital, Sichuan University, Chengdu, Sichuan 610041, China.

[3]Department of Anesthesiology, West China Hospital, Sichuan university, Chengdu, Sichuan 610041, China.

[4]National-Local Joint Engineering Research Centre of Translational Medicine of Anesthesiology, West China Hospital, Sichuan University, Chengdu, Sichuan 610041, China.

[5]Laboratory of Pulmonary Immunology and Inflammation, Frontiers Science Center for Disease-Related Molecular Network, West China Hospital, Sichuan University, Chengdu 610041, China.

*Correspondence: Jin Liu: scujinliu@gmail.com; Chan Chen: chenchan@scu.edu.cn or xychenchan@gmail.com.

[#]These authors contributed equally to this paper.

1. Supplementary Material and Methods

1.1 Quantitative real-time polymerase chain reaction

The hippocampus, prefrontal cortex and amygdala of the mice in each group were rapidly dissected and stored at -80 °C for further use. Total RNA was extracted from the above tissues *via* a TRIzol reagent kit (Invitrogen, USA). A HiScript II Q RT SuperMix for qPCR kit with gDNA wiper (Vazyme, Nanjing, China) was used for RNA reverse transcription. Afterward, the RNA purity and concentration were examined at 260/280 nm on a NanoDrop ND-1000 spectrophotometer (Thermo Fisher, USA). The sequences of the PCR primers for Lingo1 were 5'-CTTTCCCCTTCGAC ATCAAGAC-3' (forward) and 5'-CTTTCCCCTTCGACATCAAGAC-3' (reverse). The sequences of the primers for the reference gene 18S were 5'-TTGACTCAACAC GGGAAACC-3' (forward) and 5'-AGACAAATCGCTCCACCAAC-3' (reverse). The primers used were obtained from Tsingke Biotechnology Co., Ltd. (Beijing, China). Each sample was analyzed in triplicate in a 20-μL reaction containing 250 nM forward and reverse primers, 10 μL of Taq Pro Universal SYBR qPCR Master Mix (Vazyme, Nanjing, China) and 20 ng of cDNA. PCR was performed with an initial 2 min incubation at 95 °C, followed by 40 cycles at 95°C for 10 s, 55°C for 10 s, and 60°C

for 20 s, on a CFX Connect Real-Time System (Bio-Rad, USA). Relative transcript levels were calculated via the $^{\Delta\Delta}$Cq method, with 18S RNA used as a reference.

1.2 Western blot analysis

The protein from the hippocampus, prefrontal cortex and amygdala of the mice in each group was obtained *via* lysis with RIPA buffer containing a protease inhibitor cocktail (Solarbio, Beijing, China) and protein phosphatase inhibitor (Solarbio, Beijing, China) on ice for 60 min, followed by centrifugation at 12000 rpm for 10 min at 4°C. The protein concentration was quantified with a BCA protein quantification kit (Shanghai Epizyme Biomedical Technology Co., Ltd, China). The proteins were subsequently separated on polyacrylamide gels after denaturation for 10 min at 100°C. Thereafter, the separated proteins were transferred to poly(vinylidene difluoride) membranes (0.22 μm, Millipore, Bedford, USA). After being blocked with 5% nonfat milk for 60 min at room temperature, the membranes were incubated with the following primary antibodies: rabbit anti-Lingo1 antibody (Proteintech, 19097-1-AP), rabbit anti-p75NTR antibody (Proteintech, 55014-1-AP), rabbit anti-Nogo receptor antibody (Abcam, ab184556), rabbit anti-RhoA antibody (Proteintech, 10749-1-AP), rabbit anti-ROCK1 antibody (Proteintech, 21850-1-AP), rabbit anti-EGFR antibody (Proteintech, 18986-1-AP), mouse anti-Akt antibody (Proteintech, 60203-2-Ig), rabbit anti-phospho-Akt (Ser473) antibody (Proteintech, 66444-1-Ig), rabbit anti-PI3K p85α antibody (Proteintech, 60225-1-Ig), rabbit anti-phospho-PI3K p85α (Tyr607) antibody (Affinity, AF3241), rabbit anti-Tau antibody (Proteintech, 10274-1-AP), rabbit anti-phospho-Tau antibody (Proteintech, 82568-1-RR), rabbit anti-MBP antibody (Abcam, ab218011), rabbit anti-Bax antibody (Proteintech, 50599-2-Ig), rabbit anti-Bcl2 antibody (Affinity, AF6139), mouse anti-α-tubulin antibody (Proteintech, 66031-1-Ig). After overnight incubation at 4 °C, the membranes were further incubated with horseradish peroxidase-conjugated goat anti-rabbit IgG (Proteintech, SA00001-2) or goat anti-mouse IgG (Proteintech, SA00001-1) for another 60 min at room temperature. Notably, the primary antibody against phosphorylated proteins was diluted in TBST containing 3% bovine serum albumin (BSA, J&K Scientific). Moreover, the membranes used to detect phosphorylated proteins were blocked with 3% BSA in TBST. The blots were generated with the ECL Chemiluminescence Kit and visualized with an Amersham Imager 600 (Cytiva, USA) or ChemiDoc^{TM} MP Imaging System (Bio-Rad, USA).

1.3 Immunofluorescence analysis

Whole brains were excised from the mice after cardiac perfusion with 4%

paraformaldehyde (bioSharp Life Sciences, Anhui, China). After dehydration in 30% sucrose-PBS, the brains were embedded in optimal cutting temperature compound (Sakura, USA) and cut into 40 μm sections on a cryostat (Leica CM 1950, Germany). The sections were rehydrated, permeabilized and blocked with buffer containing 0.3% Triton X-100 and 10% donkey serum in PBS for 60 min at room temperature. Then, the sections were incubated with primary antibodies against Lingo1 (Proteintech, China, 19097-1-AP), MAP2 (Abcam, USA, ab92434), GFAP (Oasis Biofarm, China, OB-PGP055), Iba1 (Abcam, USA, ab283319), NeuN (Proteintech, China, 66836-1-Ig), OLIG2 (Oasis Biofarm, China, OB-PGP040), and MBP (Abcam, USA, ab218011). After overnight incubation at 4℃, the sections were subsequently immunostained with secondary antibodies (Abcam ab150175, CST 4412 and CST 8890, USA) for another 60 min at room temperature in the absence of light. After being mounted with Antifade Mounting Medium (Abcam, USA), the sections were observed and visualized under a laser scanning confocal microscope (Leica CM DMI8, Germany).

1.4 Immunohistochemistry

Whole brains were excised after cardiac perfusion with 4% paraformaldehyde, followed by postfixation overnight at 4℃. The brains were subsequently embedded in paraffin and cut into 5 μm sections. After being air-dried at 65℃ for 60 min, deparaffinized with TO reagent, rehydrated with graded alcohol solutions and antigen repair with Tris-EDTA buffer in sequence, the slices were blocked using PBS buffer that supplemented 1% goat serum and 3 % Triton X-100 for 60 min at room temperature. After that, the sections were probed with a rabbit anti-phospho-Tau antibody (Huaan Biotechnology Co., Ltd., Hangzhou, China) and then with polymer HRP-conjugated goat anti-mouse/rabbit IgG for 30 min at room temperature. After being stained with DAB and hematoxylin, the slices were mounted and observed under a VS200 microscope.

2. Supplementary Results

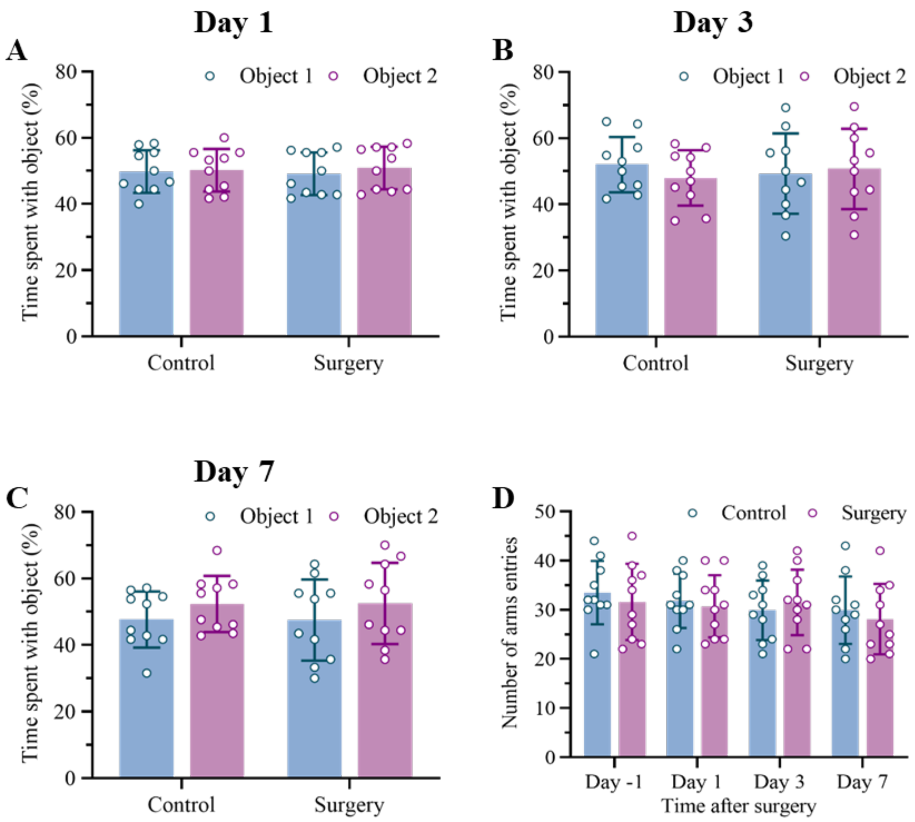

Figure S1. A-C. Exploration to the familiar objects on (A) day 1, (B) day 3 and (C) day 7 post-operation was recorded in the training phase of NOR test. D. The number of arm entries in the Y maze was recorded for the control and surgery groups on day 1 before surgery and days 1, 3 and 7 after unilateral nephrectomy. The data are presented as the mean ± standard error (n = 10).

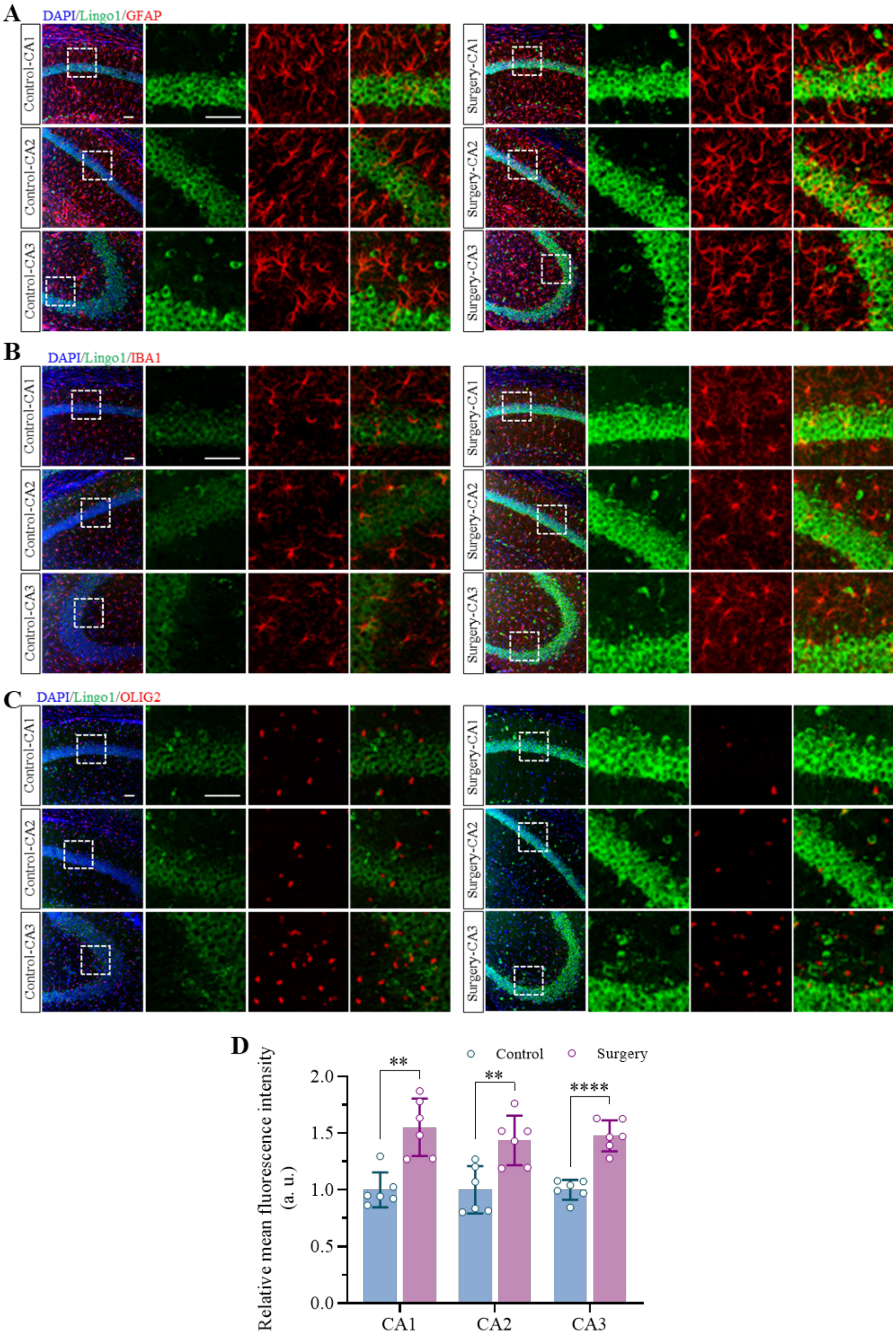

Figure S2. A-C. Representative confocal images of Lingo1 expression in astrocyte (A, GFAP), microglia (B, Iba1) and oligodendrocyte (C, OLIG2). Blue: DAPI; Green: Lingo1; Red: GFAP, Iba1 or OLIG2, Scale bar: 10 μm. D. D. Representative fluorescence images and quantification of Lingo1 expression in the hippocampal CA1, CA2, CA3 and DG regions. The data are presented as the mean ± standard error (n = 6). $^{**}P < 0.01$, $^{****}P < 0.0001$.

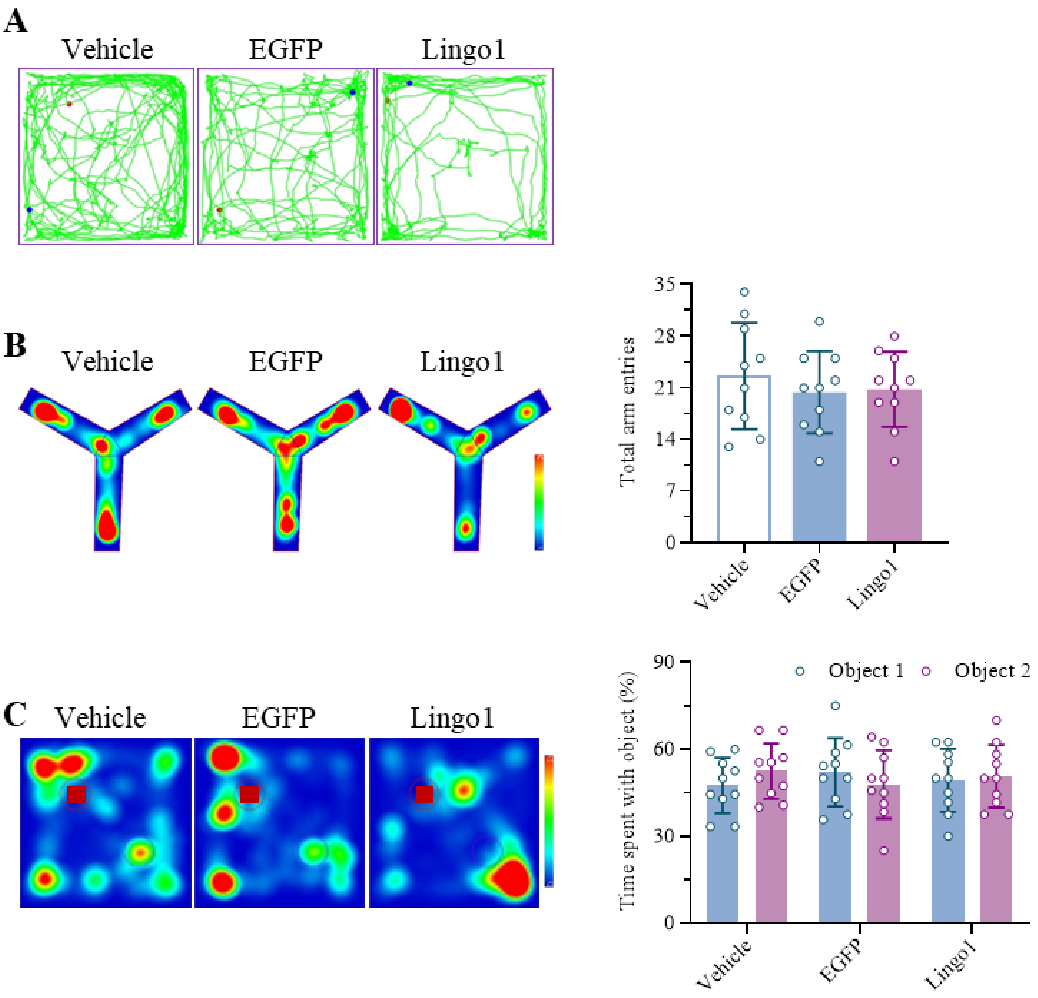

Figure S3. A. Representative trajectories of each group on day 21 after AAV-Lingo1 injection in OFT tests. B. Representative trajectory heatmap of each group on day 21 postinjection in the Y maze test, and the number of total arm entries in each group detected after AAV-Lingo1 injection for 21 days. C. Representative trajectory heatmap of each group on day 21 postinjection in the NOR test, and the exploration to the familiar objects recorded in the training phase of NOR test.

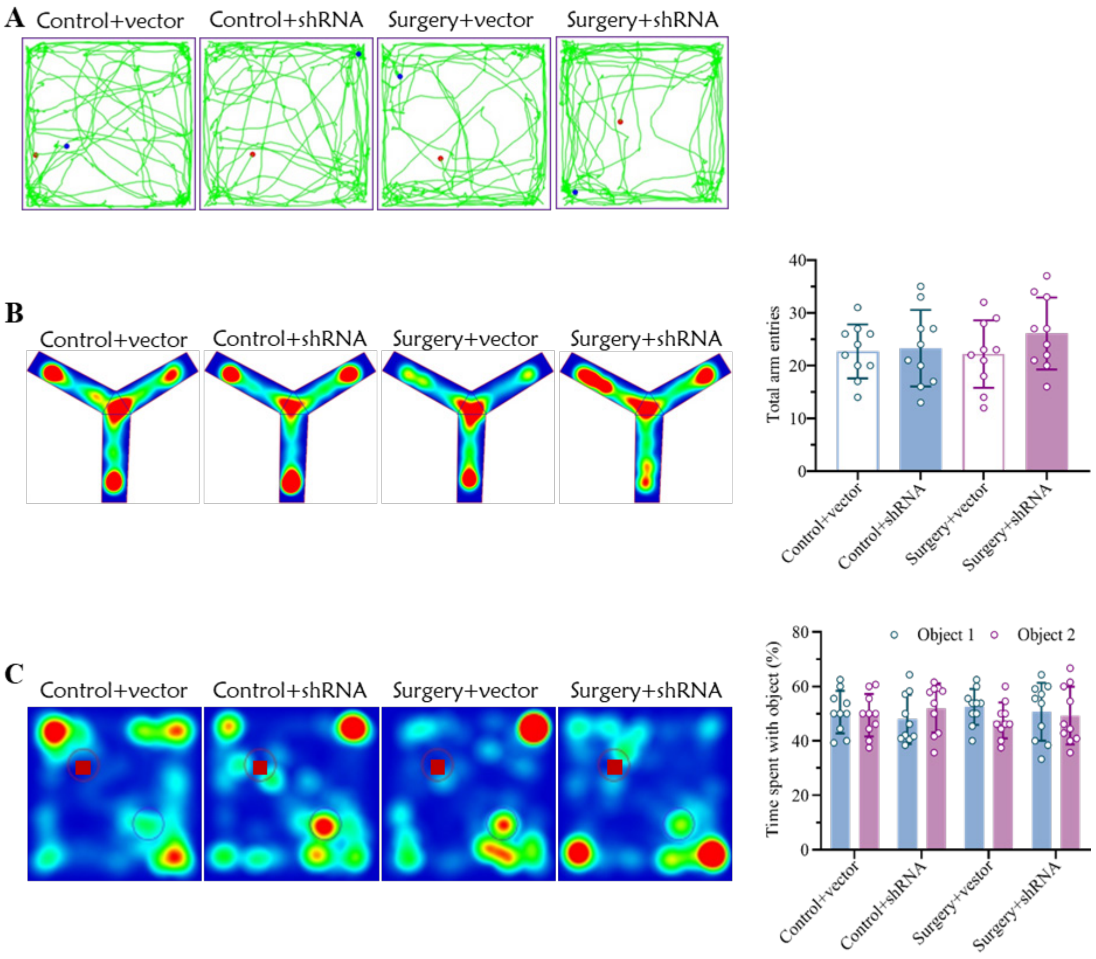

Figure S4. A. Representative trajectories of each group in OFT tests. B. Representative trajectory heatmap of each group in the Y maze test, and the number of total arm entries in each group after AAV-Lingo1 injection for 21 days, and followed by unilateral nephrectomy for 3 days. C. Representative trajectory heatmap of each group in the NOR test, and the exploration to the familiar objects recorded in the training phase of NOR test.
